# Distinct roles of tumor associated mutations in collective cell migration

**DOI:** 10.1038/s41598-021-89130-6

**Published:** 2021-05-13

**Authors:** Rachel M. Lee, Michele I. Vitolo, Wolfgang Losert, Stuart S. Martin

**Affiliations:** 1 Marlene and Stewart Greenebaum NCI Comprehensive Cancer Center, University of Maryland School of Medicine, Baltimore, MD 21201 USA; 2 Institute for Physical Science and Technology, University of Maryland, College Park, MD 20742 USA; 3 Department of Physiology, University of Maryland School of Medicine, Baltimore, MD 21201 USA; 4 Department of Physics, University of Maryland, College Park, MD 20742 USA

**Keywords:** Cellular motility, Cancer

## Abstract

Recent evidence suggests that groups of cells are more likely to form clinically dangerous metastatic tumors, emphasizing the importance of understanding mechanisms underlying collective behavior. The emergent collective behavior of migrating cell sheets in vitro has been shown to be disrupted in tumorigenic cells but the connection between this behavior and in vivo tumorigenicity remains unclear. We use particle image velocimetry to measure a multidimensional migration phenotype for genetically defined human breast epithelial cell lines that range in their in vivo behavior from non-tumorigenic to aggressively metastatic. By using cells with controlled mutations, we show that PTEN deletion enhances collective migration, while Ras activation suppresses it, even when combined with PTEN deletion. These opposing effects on collective migration of two mutations that are frequently found in patient tumors could be exploited in the development of novel treatments for metastatic disease. Our methods are based on label-free phase contrast imaging, and thus could easily be applied to patient tumor cells. The short time scales of our approach do not require potentially selective growth, and thus in combination with label-free imaging would allow multidimensional collective migration phenotypes to be utilized in clinical assessments of metastatic potential.

## Introduction

Collective migration is functionally important for metastatic dissemination, as highlighted by murine studies of multicolor tumors that lead to multicolor metastases through collective dissemination^1,2^. Individual phases of the metastatic cascade also involve collective behavior. Collective invasion has been in seen in several models, including intravital imaging of murine breast tumors^3^ and studies of ex vivo spheres from colorectal cancer patients^4^. Collective dissemination can lead to circulating tumor cell (CTC) clusters, which have up to 50 times higher metastatic potential than individual CTCs^5^. Additional evidence suggests that collective behavior plays a role during extravasation. CTC clusters pass through capillary-sized vessels^6^ and break through blood vessels intact^7^. These studies and others have identified a variety of collective behaviors, including multicellular streaming, epithelial and mesenchymal collective migration phenotypes, and expansive growth^8^, that contribute to dissemination.

This variety of dissemination behaviors may be reflective of plasticity in collective migration. Plasticity is often observed through an epithelial–mesenchymal transition (EMT) framework in which epithelial cells exhibit decreased cell–cell adhesions, lost apical–basal polarity, and modulated cytoskeletal structures^9^. Epithelial–mesenchymal plasticity (EMP), in which cells adopt a mixture of epithelial and mesenchymal features and are potentially able to interconvert between phenotypic states^9^, has been observed in many metastasis studies. Breast cancer cells that co-express both epithelial and mesenchymal markers are more tumorigenic than cells which exhibit epithelial or mesenchymal phenotypes^10^. Cells exhibiting EMP are a major source of metastasis formation^11^. Increased EMP was also found in CTC clusters isolated from blood samples from breast cancer patients^12^. Decreases in cell–cell adhesion during EMP may give cells the flexibility to respond to microenvironmental cues and to transition between collective and individual migration as needed^13^. Plasticity in collective behavior is also seen through the framework of an unjamming transition, in which epithelial monolayers flow collectively without necessitating the activation of EMT-transcription factors or expression of mesenchymal markers^14^. Signatures of unjamming have been observed in both non-tumorigenic MCF10A cells and in breast cancer models^15,16^. Unjamming also allows mesenchymal tumor cells to adapt to their surroundings and migrate collectively^17^.

Despite the importance of collective migration during metastatic dissemination, the connections between oncogenic mutations, in vivo tumor outcomes, and collective behavior are unclear. Here we use a genetically defined breast cancer model system to connect oncogenic mutations with a quantitative collective phenotype. In particular, we focus on phosphatase and tensin homologue (PTEN) loss, which is found in 24% of breast cancers^18^. The phosphatidylinositol 3 kinase (PI3K) pathway, which includes PTEN, has alterations in over 70% of breast cancer patients^18^, and PTEN loss has been associated with poor outcomes in breast cancer patients^19^. Our model system uses overexpression of KRas(G12V), a frequent cancer driver^20,21^, alone and in combination with PTEN loss to study the response to PTEN loss in a non-tumorigenic and an activated oncogenic background. These cell lines have characterized in vivo tumorigenicity^22^ and metastatic potential^23^ and exhibit distinct migration behavior when individual cells are allowed to move within microfluidic channels^23^. Here we adapt a multidimensional quantitative collective migration phenotype^24–26^ to this breast cancer model system to measure the impact of oncogenic mutations on collective behavior. We find that PTEN loss and activated KRas overexpression have opposing effects on migration and that the decreased collective behavior of activated KRas overexpression dominates the migration phenotype. Quantitative phenotypes allow us to investigate signatures of collective migration that are associated with the known in vivo tumor outcomes of our model system and determine metrics that could provide insight into tumorigenicity without requiring the selective pressures associated with long term growth in culture.

## Results

### Collective migration changes during cancer progression

We first compare non-malignant MCF10A mammary epithelial cells to the aggressively metastatic MDA-MB-231 breast cancer cells. Cells were plated in circular cell sheets which were confluent in the center and surrounded by empty space (Supplementary Figure S1). In contrast to other techniques, such as scratch assays or the removal of a physical barrier to prompt migration^27,28^, the preparation of these circular cell sheets does not include the potentially complicating factors of a physical stimulus inducing cell death or disruption of the migration surface.

**Figure 1:**
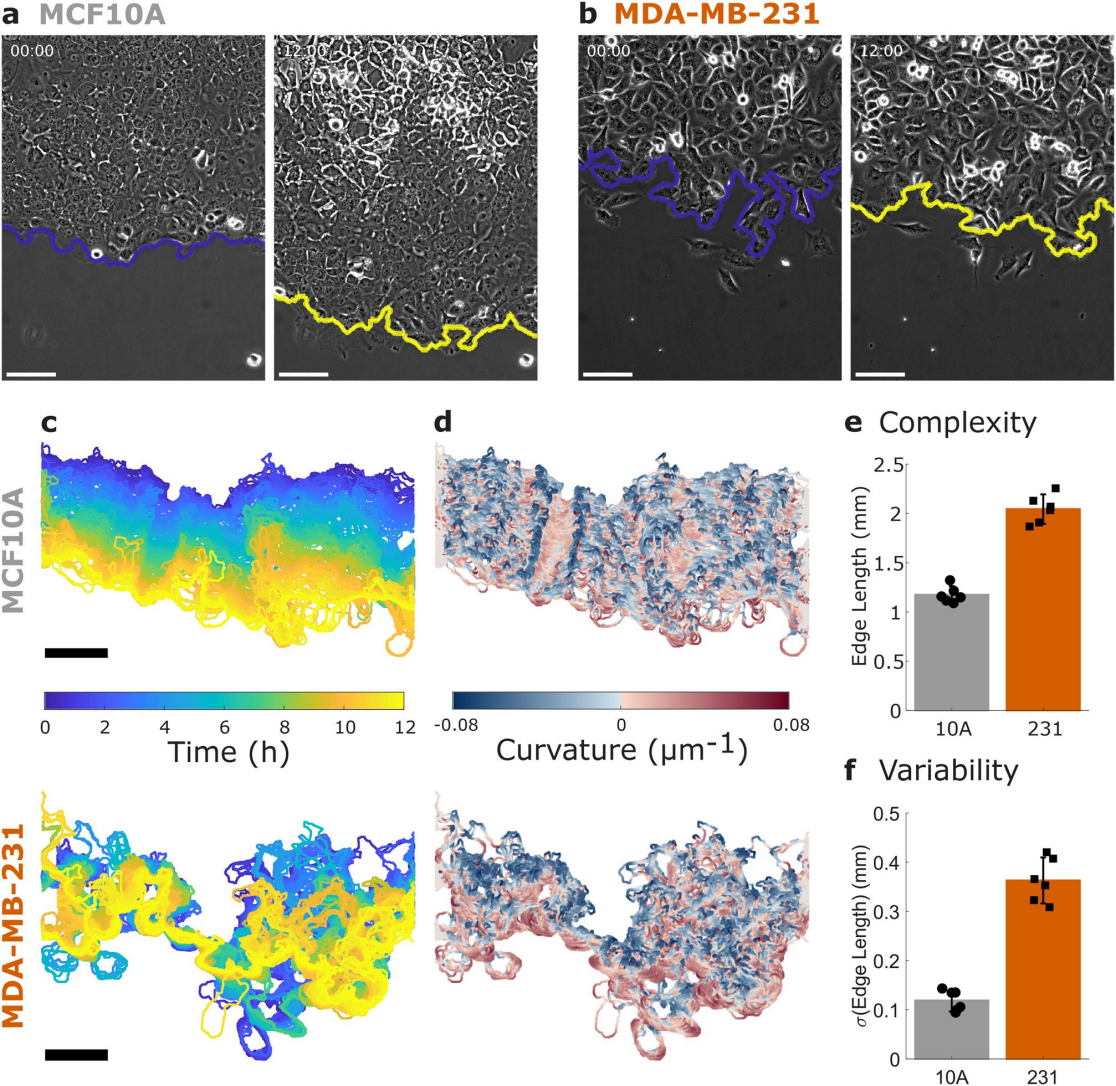
Collective Migration Changes During Cancer Progression. (**a**) Non-tumorigenic MCF10A cells and (**b**) metastatic MDA-MB-231 cells migrate in a collective migration assay over 12 h. The cell sheet leading edge is indicated by a blue (initial) or yellow (12 h) line and scale bars are 100 µm. See also Supplementary Video S1. (**c**) The dynamics of the leading edge are collective in the MCF10A cells (top) but disordered in the MDA-MB-231 cells (bottom). (**d**) Coloring the leading edge by curvature shows persistence of local features in the MCF10A cells (top) and disorder in the MDA-MB-231 cells (bottom). (**e**) Edge length quantifies the complexity of the leading edge. (**f**) Variability in edge length over time quantifies the dynamics of the leading edge. N = 6 independent experiments. Error bars indicate 95% confidence intervals.

To investigate the behavior of these cell sheets over time, regions of interest (ROIs) near the top and bottom edge were imaged over 12 h. Example images of an MCF10A cell sheet (Fig. 1a) and an MDA-MB-231 cell sheet (Fig. 1b) are shown for the start and end of imaging (12 h). To investigate dynamic features behavior in the cell sheet, images were taken every three minutes, as shown in Supplementary Video S1.

An illustration of cell sheet dynamics is shown in Fig. 1c, where the leading edge of the sheet is colored by time for the cell sheets in Fig. 1a-b. The smooth transition from blue (0 h) to yellow (12 h) indicates that the leading edge of the MCF10A sheet progresses steadily forward, while the MDA-MB-231 edge shows disordered progression. Coloring each edge by time (Fig. 1c) indicates displacement, while coloring the edge by local curvature (Fig. 1d) emphasizes the persistence of local features. In the MCF10A sheet, persistent regions of blue and red (i.e. high curvature) progress forward over time, while the MDA-MB-231 edge does not show consistent features. The length of the leading edge reflects the complexity of bends and turns across the image. Comparing the average edge length (Fig. 1e), we find that the MDA-MB-231 edge is on average 0.9 mm longer than that of the MCF10A. Compared to the minimum edge length of approximately 0.6 mm (the imaging ROI width), an increase of 0.9 mm indicates a substantial increase in complexity. We also investigate the variability of edge length over time (Fig. 1f) and find that the MDA-MB-231 cells have approximately three times the variability of the MCF10A cells.

### Collective behavior quantitatively decreases in metastatic cells

**Figure 2:**
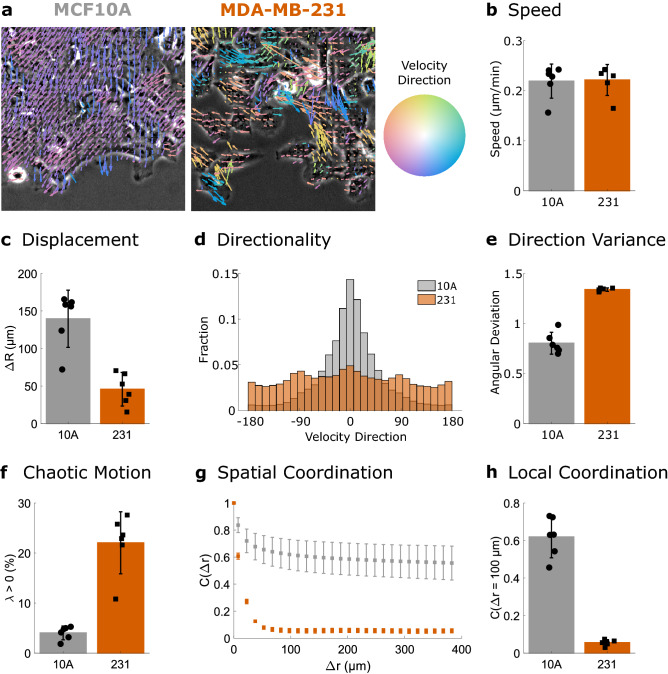
Collective behavior quantitatively decreases in metastatic cells. (**a**) PIV flow vectors colored by motion direction and overlaid on images of MCF10A cells (left) and MDA-MB-231 cells (right). (**b**) Mean speed of the PIV flow field. (**c**) Displacement of the leading edge. (**d**) Cumulative distribution across replicates of velocity direction. (**e**) Variability in velocity direction quantified by angular deviation. (**f**) Chaotic motion quantified by the percentage of positive finite-time Lyapunov exponents (λ). (**g**) Spatial coordination across length scales quantified by the autocorrelation of radial velocity. (**h**) Local coordination is defined as the correlation value at 100 µm (a few cell lengths). N = 6 independent experiments. Error bars indicate 95% confidence interval.

To further analyze the collective dynamics, we use Particle Image Velocimetry (PIV) to measure flows within the cell sheet. Example flow fields for the MCF10A and MDA-MB-231 cells are shown in Fig. 2a. Speeds calculated from PIV do not show differences between the MCF10A and MDA-MB-231 cells (Fig. 2b), despite the differences in leading edge displacement illustrated in Fig. 1 and quantified in Fig. 2c.

This difference is reconciled by measurements of directionality within the cell sheet. As shown by the vector colors in Fig. 2a, the MCF10A cells flow into the space at the sheet edge, while the MDA-MB-231 cells migrate in multiple directions. Distributions of velocity direction (Fig. 2d) emphasize differences in directionality between the cell lines. This is further quantified by angular deviation (Fig. 2e); the higher values in the MDA-MB-231 cells indicate less directional migration.

Additional collective migration metrics can provide insight into which features of migration are most affected by perturbations^24,26^, thus we measured collective behavior over long length and time scales. Directionality in Fig. 2d-e is measured on the scale of the imaging (minutes). Finite time Lyapunov exponents (λ), however, are a measure of the divergence of nearby trajectories over long time scales (in this case, 2 h). Positive λ are associated with chaotic flows and thus increased positive λ values (Fig. 2f) indicate decreased coordination in the MDA-MB-231 cells over long timescales.

To measure coordination of the cell sheet over long distances, we compute the spatial autocorrelation of the radial velocity (Fig. 2g). The MCF10A cells show similarity with neighbors hundreds of µm away, while the MDA-MB-231 cells show decreased correlations after roughly 50 µm. To quantify coordination with local neighbors, we compare the autocorrelation values at 100 µm (Fig. 2h), which show higher spatial coordination in the MCF10A cells.

### Activated KRas and PTEN^−/−^ have opposing effects on collective migration

**Figure 3:**
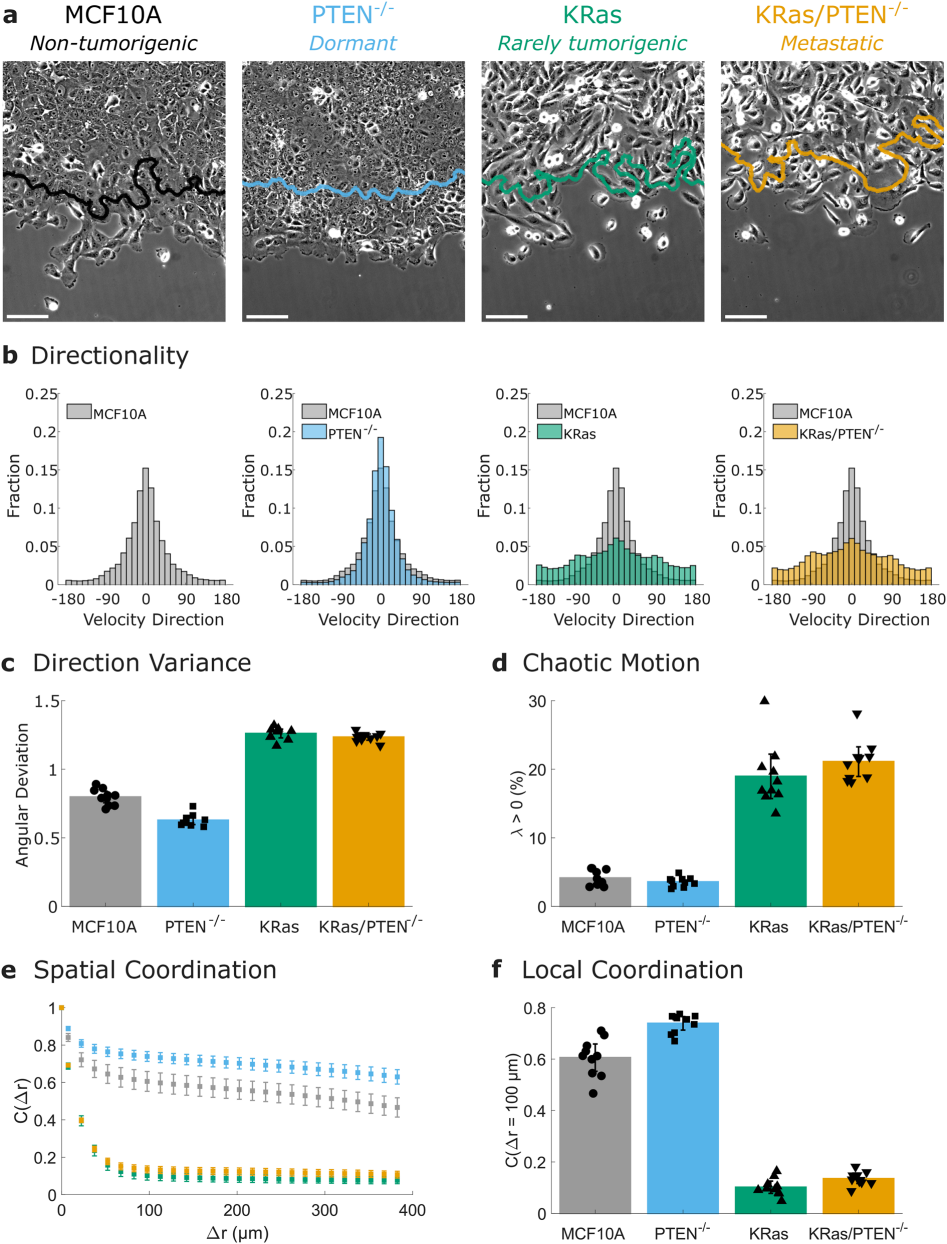
Activated KRas and PTEN^−/−^ have opposing effects on collective migration. (**a**) Images from collective migration assays on cells from a genetically defined cancer model system after 12 h of migration. The position of the initial (t = 0 h) leading edge is indicated by the colored line and scale bars are 100 µm. See also Supplementary Video S2. (**b**) Cumulative distributions of velocity direction. (**c**) Variability in velocity direction quantified by angular deviation. (**d**) Chaotic motion in the cell sheet quantified by the percentage of positive finite-time Lyapunov exponents (λ). (**e**) Spatial coordination across length scales quantified by the autocorrelation of radial velocity. (**f**) Local coordination is defined as the correlation value at 100 µm (a few cell lengths). N = 10 independent experiments. Error bars indicate 95% confidence interval.

Our finding that the MDA-MB-231 cells exhibit a less collective phenotype than the MCF10A cells is in agreement with previous studies which have found disordered collective behavior in cancer cells^15,27^. Next we investigated the collective behavior of a genetically defined model system which has been characterized in vivo for primary tumorigenesis and metastatic efficiency^22,23^. This model allows us to connect our multidimensional phenotype to mutations with known tumor outcomes. The model system is based on the MCF10A cells, which do not form tumors in murine models. PTEN deletion allows the cells to remain dormant in vivo, while KRas activation leads to dormancy or, rarely, to tumor formation^22^. However, when the two mutations are combined, KRas/PTEN^−/−^ cells form aggressive primary tumors^22^ and lung metastases^23^. Images of this model system migrating collectively are shown in Fig. 3a and Supplementary Video S2. As seen in Fig. 3a and quantified in Supplementary Fig. S2, cell lines with overexpressed active KRas show increased complexity in their leading edge.

PIV analysis indicates that the PTEN^−/−^ cells are slightly more directional than the MCF10A control, while both the KRas and KRas/PTEN^−/−^ cells are less directional (Fig. 3b). The KRas and KRas/PTEN^−/−^ cells both show increased angular deviation values (Fig. 3c) compared to the MCF10A, in a similar manner as the MDA-MB-231 cells (Fig. 2e). This angular deviation increase is reflected in the decreased progression of the leading edge as illustrated in Fig. 3a and quantified in Supplementary Fig. S3. The KRas and KRas/PTEN^−/−^ cells show increased chaotic motion compared to the MCF10A control (Fig. 3d) as well as decreased spatial coordination (Fig. 3e-f). Chaotic motion in the MCF10A cells is largely localized to the leading edge of the monolayer (Supplementary Fig. S4). This increase in chaotic motion at the leading edge is diminished in the PTEN^−/−^ cells (Supplementary Fig. S4), which show a significant decrease in chaotic motion compared to the MCF10A cells for 0.95 < r/R < 1. The PTEN^−/−^ cells also show the opposite trend of increased spatial coordination and thus trend towards increased collective behavior in multiple metrics.

As seen in Supplementary Video S2, cells undergo proliferation during the 12 h of migration. Prior published work on our genetically defined model system has shown these four cell lines have similar growth rates in normal cell culture medium but show differences during growth in serum starved conditions^22^. Thus, conducting our migration assays in normal culture medium (5% serum) allows for a similar amount of proliferation in each cell line. Indeed, measurements of cell density in the migrating sheets show similar increases in cell number and similar average densities across all four cell lines (Supplementary Figures S5-6). Because published proliferation rates and measured densities are not significantly different in these cell lines, the changes in collective behavior we see with PTEN deletion and KRas activation are not due to differences in proliferation or density between the cell lines.

### Activated KRas dominates the collective migration phenotype

**Figure 4:**
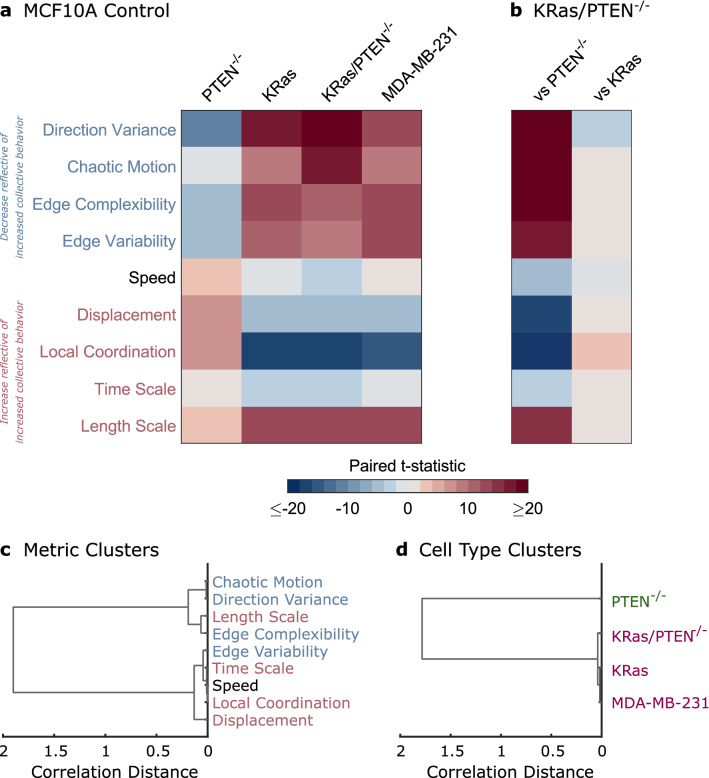
Activated KRas dominates the collective migration phenotype. (**a**) Multidimensional phenotype for four cell lines as compared to the MCF10A control. The strength of changes in metrics are indicated by color representing the paired t-statistic (see Supplementary Fig. S7). (**b**) Migration phenotypes comparing the KRas/PTEN^−/−^ cells to the single mutant cell lines. (**c**) Clustering of migration metrics using correlation distance. (**d**) Clustering of cell lines using correlation distance.

To compare multiple metrics with varied units of measurement, we convert the results shown in Figs. 1, 2 and 3 into paired t-statistics (see Supplementary Fig. S7). The multidimensional phenotype can then be summarized as shown in Fig. 4a. Migration metrics, shown on the y-axis, are colored by whether the metric would be expected to decrease (blue) or increase (red) as collective behavior increases. The cell lines shown on the x-axis are compared to the MCF10A cells as a control. Figure 4a shows that while the PTEN^−/−^ cells tend to increase in collective behavior, the KRas, KRas/PTEN^−/−^, and MDA-MB-231 cells show decreased collective behavior. In Fig. 4b, the KRas/PTEN^−/−^ cells are compared to the individually mutated cell lines; the higher similarity with the KRas cell line emphasizes the dominant nature of KRas on the collective migration phenotype.

Clustering of the migration metrics based on correlations within the cell lines (Fig. 4c), reveals that the metrics largely separate as would be expected into groups which either increase (red) or decrease (blue) as collective behavior increases. One deviation from this trend, the characteristic motion length scale, could reflect both changes in cell size and in spatial coordination. Clustering of the cell lines leads to two clusters: the highly collective PTEN^−/−^ cells and three cell lines with disordered migration (Fig. 4d).

## Discussion

Using a quantitative multidimensional phenotype, we find that two tumor-associated mutations (PTEN loss and activated KRas overexpression) have distinct effects on behavior. PTEN deletion leads to an increase in collective behavior compared to the MCF10A cell line. In contrast, KRas(G12V) overexpression leads to a clear decrease in collective behavior, similar to the decrease in collective behavior observed in the highly metastatic MDA-MB-231 cell line. The KRas/PTEN^−/−^ cell line also shows decreased collective behavior, suggesting that KRas dominates the collective migration phenotype and that the effects of the mutations are not additive. This is in agreement with the known KRas mutation (G13D) in the MDA-MB-231 cell line^29^, which is often used a model system for rapid metastasis. Although Ras mutations are rare in breast cancer, KRas is one of the most frequently altered genes across tumor types^20,21^, persistent activation of Ras is seen in many breast cancer cell lines^30^, and components of the Ras signaling pathway (e.g. ERK) are mutated or highly activated in breast cancer^31,32^. This signaling pathway has also been implicated in collective dissemination. A recent study of an MCF10A-based model of unjamming in which the small GTPase RAB5A was overexpressed lead to collective invasion^16^. This result was mediated by ERK activation, supporting the idea that pathways downstream of Ras may play an important role in collective breast cancer dissemination.

Although KRas dominates the measured collective phenotype, this mutation alone is not sufficient for aggressive tumorigenicity^22^ or metastasis^23^ in vivo. In vitro studies have also found that Ras activation is not sufficient for breast cancer cells to have invasive properties^30^. The trend toward increased collective behavior observed in the PTEN^−/−^ cells raises the possibility that the opposing migratory phenotypes of PTEN^−/−^ and KRas may provide additional plasticity to the double mutant line and enable EMP in response to in vivo microenvironments. Microenvironments encountered during dissemination can include changes in stiffness or texture that are not captured by our in vitro migration assay^33,34^. Cells that have undergone an unjamming transition or that exhibit EMP are able to migrate collectively in response to in vivo microenvironmental cues^13,17^. EMP may be especially relevant during the circulation phase of metastasis, where EMP signatures such as E-cadherin expression are required for successful collective dissemination^35^. PTEN loss may thus allow cells overexpressing KRas the additional flexibility necessary to adapt to in vivo cues.

PTEN loss may also impact other metastasis pathways. Migration and invasion have been recognized as a central hallmark of cancer^36^, but there are many other transformations that contribute to cancer progression. PTEN loss leads to growth-factor independent proliferation, as well as likely resistance to apoptosis^37^. The apoptotic insensitivity conferred by PTEN loss may play a role in our breast cancer model system in light of previous results showing that apoptotic resistance does not directly promote tumor growth, but can increase metastasis when combined with activation of the Ras-MEK pathway^38^. The need for additional information to distinguish the metastatic potential of the KRas and KRas/PTEN^−/−^ cell lines is also in agreement with the observed individual migration of these cell lines. Individual migration was found to be highly predictive of metastatic potential, but adding Ki-67 as a proliferation marker allowed for the removal of false positives and helped distinguish cell lines^23^. We note that our migration assays were completed in full-serum culture media, which is a condition in which the cell lines of the genetically defined cancer model system studied here were previously found to have the same growth rate^22^. Thus, our collective migration phenotypes are measured under conditions where growth is consistent between the cell lines, which allows inherent differences in collective behavior to be measured. However, by controlling for cell proliferation, our collective migration system likely does not capture the effects on collective migration of changes in proliferation or cell death that could be prompted by more complex microenvironmental conditions.

Markers such as Ki-67 are already used clinically for breast cancer prognosis. Increasing evidence suggests that migration may be more predictive of outcome than growth^23,39^. Migration phenotypes have also been used to identify novel targets of dissemination that may not have been identified by screens of growth alone^40^. This suggests that adding assessments of collective behavior such as the quantitative phenotype described here may allow for more accurate clinical assessments of metastatic potential. The migration assay employed here does not require cell growth and thus avoids selective pressures associated with long-term culture of patient tumor samples in vitro or in mouse models. This assay also uses relatively simple phase-contrast microscopy and does not require fluorescent labeling. The 12 h time course analyzed here allows for the measurement of metrics on multiple length and time scales that can help distinguish cells which behave similarly on shorter time scales^24^. As metastasis occurs over days and weeks in vivo, measurements of dynamic migration behavior over hours adds depth to cancer phenotypes. However, several of the metrics in our multidimensional phenotype are on the scale of minutes (direction variance, edge complexity, speed, local coordination, and length scale) and could thus be applied to shorter time courses to allow for faster assessment of collective behavior.

Our multidimensional phenotype integrates data on a range of length and time scales, and thus captures effects from a broad range of biophysical processes. Given the importance of migratory phenotypes for metastatic potential, our analysis could be incorporated into tools to provide rapid assessments of patient cells on the scale of hours, compared to the days to weeks required for spheroid growth or the months required for PDX models. Indeed, both directionality and spatial coordination show strong decreases in the KRas cell lines and can be calculated from as few as two images taken 3 min apart. Continuing the label-free imaging over a period of at least 2 h allows for calculation of chaotic motion, which creates a more robust multidimensional phenotype.

Using this multidimensional phenotype, we find that the decrease in collective behavior associated with KRas activation dominates over the increased collective migration effects of PTEN loss. Cancer metastasis is a complex phenotype and collective migration assessments alone are not sufficient to capture the differences in metastatic potential of the KRas and KRas/PTEN^−/−^ cells. We propose that PTEN loss could drive additional hallmarks of cancer which could be assessed in combination with collective migration phenotypes. For example, enhanced cell proliferation may be detected with clinical markers, such as Ki-67^23^, while enhanced plasticity may be directly tested through in vitro microenvironmental cues, such as texture^41–43^. Quantitative assessments of migration allow for the investigation of the role of specific oncogenic mutations in collective dissemination as well the impact of drug treatments^26^. This creates opportunities for future studies to identify potential targets of collective dissemination in vivo and to also identify the role current treatments may play in curtailing or unintentionally enhancing^44^ metastatic progression.

## Methods

### Experimental design

Biological replicates were imaged in a complete block design with comparisons made between cell sheets imaged in the same 12 well plate (see *Clustering Analysis*). Phase contrast images of the cell sheets (*see Migration Assay and Imaging*) were analyzed using a particle image velocimetry (PIV) analysis pipeline (see *Image Analysis*). Blinding was not performed for this study; all image analysis was conducted automatically with the same sets of analysis parameters.

Sample size was determined by performing a power analysis on previously collected migration data assuming a paired t-test. At a threshold of 90% power, N = 10 biological replicates (used for comparing the genetically defined mutants) were sufficient to resolve a 0.1 difference in angular deviation. Based on the prior literature, a large difference was expected between the MCF10A and MDA-MB-231 cells, thus N = 6 replicates were used, which the power analysis suggested were sufficient to resolve a 0.15 difference in angular deviation values. Biological replicates (independent experiments conducted on separate experimental days) were composed of two technical replicates (duplicate wells in the same 12 well plate); technical replicates were averaged for further analysis. No data or outliers were excluded.

### Cell lines and culture

All cell lines used in this study are immortalized human breast epithelial cells. MCF10A cells (ATCC) were maintained in DMEM/F12 medium (Thermo Fisher Scientific 11-330-057) with 5% horse serum (Thermo Fisher Scientific 26,050,088) that was supplemented with 10 μg/ml insulin (ThermoFisher Scientific 12,585,014), 10 ng/ml EGF (PeproTech AF-100-15), 0.5 μg/ml hydrocortisone (Sigma-Aldrich H4001), and 100 ng/ml cholera toxin (Sigma-Aldrich C8052). MDA-MB-231 GFP-LUC cells^22,23^ were maintained in DMEM (Corning 10-017-CV) with 10% FBS (Gemini Bio-Products 100-106). MCF10A, MCF10A PTEN^−/−^^37^, MCF10A KRas(G12V)^22^, and MCF10A KRas(G12V)/PTEN^−/−^^22^ cells were infected with a LifeAct-GFP expressing lentivirus; LifeAct cells were used in all comparisons between these four cell lines reported in this work. All LifeAct cells were cultured in MCF10A media supplemented with 0.5 μg/ml puromycin (AG Scientific P-1033-sol). All cells were maintained in a humidified atmosphere at 37 °C and 5% CO_2_. All cells used in this study tested mycoplasma negative using the Lonza MycoAlert (VWR 75870-454) testing system.

### Migration assay and imaging

Glass bottom 12-well plates (MatTek Corporation P12G-1.5-14-F) were coated with 3.25 μg/cm^2^ collagen IV (Corning 354,233). The plate was placed on ice and 500 ml of collagen IV solution (3.25 μg/cm^2^ collagen IV in 0.05 M HCl) was added to each well for 1 h before rinsing each well twice with water. Cells for the migration assay were suspended at a concentration of 1.5 × 10^6^ cells/ml and plated as a 5 µL drop in the center of each well. After approximately 45 min at 37 °C, non-adherent cells were removed by medium rinses, the well was filled with 1 ml medium, and the cells were allowed to adhere in the incubator overnight. This resulted in a roughly circular sheet of cells surrounded by empty space.

Approximately 1 h before the start of imaging, the medium in each well was replaced with 1 ml of fresh medium. Cells were imaged overnight on a PerkinElmer UltraView VoX spinning-disk confocal microscope. Before time-lapse imaging, a single snapshot of the entire cell sheet was recorded by tiling multiple phase contrast images. A motorized x–y stage and Nikon Perfect Focus System were used to acquire images at multiple positions over time. The microscope was equipped with a Tokai Hit environmental chamber with a humidity reservoir and was set to 37 °C and 5% CO_2_. Images were taken using a 10 × phase-contrast objective (Nikon CFl Plan Fluor, NA 0.3). Images were collected using PerkinElmer’s Volocity software (version 6.4.0) with a Hamamatsu C10600-10B ORCA-R2 camera that recorded 12-bit images (1024 pixels × 1344 pixels, 0.582 µm/pixel). Images were recorded every 3 min for 12 h.

### Image analysis

The leading edge of each migrating cell sheet was segmented using custom MATLAB code^45^ as previously described^24^. Sobel filtering, median filtering, and morphological operators were used to find a binary image that indicated the location of the cell sheet. Dijkstra’s algorithm, as implemented by Sebastien PARIS for MATLAB^46^ was used to find the coordinates of the leading edge. Two opposing edges of each cell sheet were imaged over time; these two edges were fit to a circle to calculate the radius of the approximately circular cell sheet over time. The two edges combined with the microscope stage positions from overnight imaging were used to create a polar coordinate system for each cell sheet to define the radial direction of motion (the direction of motion expanding the sheet) as previously described^24^.

PIV analysis was based on the MatPIV toolbox by Kristian Sveen (GNU general public license)^47^. This toolbox was used to extract velocity information from the time-lapse images as previously described^24,25^. Briefly, two first-pass calculations with 64 pixel × 64 pixel windows (approximately 37 µm × 37 µm) were followed by two iterations of 32 pixel × 32 pixel windows, in all cases with a 50% overlap. Outliers were detected using a signal-to-noise filter (threshold of 1.3).

### Migration analysis

The length of the segmented cell sheet edge was calculated as the summed distance between all boundary points. As all leading edges spanned the same image width, the mean edge length over time was used as a measure of the edge complexity. The standard deviation of the edge length over time was reported as the edge variability. The difference between the calculated cell sheet radius at *t* = 0 h and *t* = 12 h was reported as the cell sheet displacement. Curvature of the edge was calculated as the Menger curvature of points within 40 boundary points of the boundary point of interest.

Bar graphs of speed report the mean speed of PIV vectors averaged over both time and space. Direction distributions indicate the direction of PIV vectors with respect to the radial direction of motion and are shown as cumulative distributions across biological replicates. The variability in velocity direction was parametrized by the angular deviation, calculated as $\sqrt{2(1-z)}$ where *z* is defined in Eq. (1). This equation bounds the values of angular deviation between zero (no variance in directionality) and $\sqrt{2}$ (highly varied directionality).

1 $$z= \frac{1}{N}{\left[{\left(\sum_{i}^{N}\mathrm{cos}{\theta }_{i}\right)}^{2}+{\left(\sum_{i}^{N}\mathrm{sin}{\theta }_{i}\right)}^{2}\right]}^{1/2}$$

Finite-time Lyapunov exponents (λ) were calculated by computationally tracing virtual particles through the PIV flow field. As previously described, we use a deformation time of 2 h^25^ to allow the λ values to asymptotically approach Lyapunov exponents^48^. Tracer particles are initiated on the PIV grid but allowed to move off the grid as the flow field evolves over time. For each set of four virtual particles that were initially neighbors, the logarithm of the largest eigenvalue of the Cauchy–Green strain tensor is the local λ value. Positive λ values indicate an exponential sensitivity to initial conditions and are associated with chaotic flow fields, thus the percentage of positive λ values is used as a measure of the chaotic motion in the system.

Spatial autocorrelations were calculated for the radial velocity component of the PIV flow field, *v*, using Eq. (2). The value of the autocorrelation at Δ*r* = 100 µm was used as a metric of local coordination within the cell sheet.

2 $$C\left(\Delta r\right)= \frac{\sum_{{r}_{i}}v({r}_{i})\bullet v({r}_{i}+\Delta r)}{\sqrt{\sum_{{r}_{i}}{v}^{2}({r}_{i})\bullet \sum_{{r}_{i}}{v}^{2}({r}_{i}+\Delta r)}}$$

To calculate characteristic length and time scales of motion from the PIV flow fields, we used a coarse graining technique described by Zorn et al.^49^. By averaging the PIV flow field over increasing time intervals, the variance of the flow field decreases. This variance can be fit to an exponential decay as shown in Eq. (3) to calculate the characteristic time scale *t*_*c*_. The second term of this equation accounts for the finite accuracy of the microscope stage in returning to a position during multi-position imaging (*t*_*imaging*_ = 3 min).

3 $$\sigma =A{e}^{-t/{t}_{c}}+\frac{B}{\sqrt{t/{t}_{imaging}}}+C$$

Similarly, the characteristic length scale of motion was calculated by averaging the PIV flow field over increasing spatial distances. The decreasing variance in the flow field can then be fit to the exponential decay in Eq. (4) to calculate the characteristic length scale *l*_*c*_.

4 $$\sigma =A{e}^{-l/{l}_{c}}+B$$

For plots of migration metrics versus *r/R*, *R* is the radius of the cell sheet, while *r* is the location within the cell sheet. Data was binned in radial sections of size 0.05 *r/R* for plotting.

### Density calculations

To measure density in the cell sheets, duplicate 12-well plates of cell sheets were created as described above in *Migration Assay and Imaging*. After the cell sheets were allowed to adhere overnight, the medium in each well was replaced with 1 ml of fresh medium. One hour later, one of the plates was fixed in ice cold methanol for 10 min on ice, followed by two rinses with PBS. The second plate was fixed 15 h after the first plate in the same manner. Both plates were then stained with DAPI. DAPI-stained plates were imaged on a PerkinElmer UltraView VoX spinning-disk confocal microscope with the same configuration as described above. DAPI images were illuminated with an Intensilight mercury light source.

The shape of each cell sheet was segmented using the edge segmentation approach described in *Image Analysis*. Nuclei centroids were found using a peak-finding algorithm by Crocker and Grier^50,51^. Images were bandpass filtered with a lower bandpass of size 4 pixels and a higher bandpass of size 30 pixels. A threshold of 6 was used for the peak finding function *pkfind*. False positives in the cell sheet boundary (caused by collagen clumps in the phase contrast image) were removed by excluding regions of the cell sheet region that did not contain nuclei. The cell sheet boundary was fit to a circle to calculate the radius of the cell sheet. Cell number fold change was calculated as the number of counted nuclei at t = 15 h divided by the number at t = 0 h. Average density was calculated as the number of cells within the cell boundary divided by the area of the cell sheet region. Density as a function of *r/R* was calculated by dividing the number of cells in a radial section of size 0.05 *r/R* by the area of the radial section.

### Clustering analysis

Migration assay imaging data was collected as paired biological replicates where multiple cell lines were imaged in the same 12-well plate over time using multi-position time-lapse imaging. To compare multiple metrics in a multidimensional collective migration phenotype, paired t-statistics were calculated for each metric. As shown in Supplementary Fig. S7, a paired difference was first calculated for each cell line compared to the control. The mean of this paired difference divided by the standard error of the paired difference is the paired t-statistic, which is used to report summaries of multidimensional migration phenotypes.

Clustering of cell lines and migration metrics was based on the correlation distance (one minus the sample correlation between points). An agglomerative hierarchical cluster tree was calculated using MATLAB’s linkage function using the ‘average’ method for the ‘correlation’ distance metric between clusters. Dendrograms were created using the leaf ordering determined by MATLAB’s optimalleaforder function.

The cluster tree was divided into distinct clusters as previously described^26,52^. Clustering matrices for the metrics and cell lines respectively were ordered according to the optimal leaf order. This matrix was then fit to a block-diagonal structure using greedy algorithm for splitting the data into blocks and a Bayesian information criterion (BIC) to avoid overfitting in determining cluster boundaries.

### Statistical analysis

For each metric, means shown in the respective figures were calculated from N = 6 (MDA-MB-231) or N = 10 (genetically defined model system) independent experiments. Confidence intervals on the mean were calculated as the standard error of the mean (standard deviation divided by the square root of the number of experiments) multiplied by the appropriate critical t-value. Critical t-values were calculated from an inverse t-distribution using a two-tailed alpha of 0.05. Confidence intervals were not adjusted for multiple comparisons. All underlying data for the presented means and confidence intervals is presented in tabular format in Supplementary Dataset S1.

## Supplementary Information

Supplementary Information 1.

SuppSupplemental Dataset S1.

Supplementary Video 1.

Supplementary Video 2.

## Data Availability

Figure source data is available in Supplementary Dataset S1. The code used for PIV and collective migration analysis is available from https://github.com/ScientistRachel/multiScalePIVanalysis; 10.5281/zenodo.4718746.
