# Supplementary material for: Distinct roles of tumor associated mutations in collective cell migration
Source: Sci Rep. 2021 May 13;11:10291. doi: 10.1038/s41598-021-89130-6 (PMC8119502; doi:10.1038/s41598-021-89130-6)
Supplement: Supplementary file 1 — Supplementary Information 1. [file 41598_2021_89130_MOESM1_ESM.pdf]

SUPPLEMENTARY INFORMATION

Distinct Roles of Tumor Associated Mutations in Collective Cell Migration

Rachel M. Lee[1,2], Michele I. Vitolo[1,3], Wolfgang Losert[3,4], Stuart S. Martin*[1,3]

[1]Marlene and Stewart Greenebaum NCI Comprehensive Cancer Center, University of Maryland School of Medicine, Baltimore, MD 21201, USA
[2]Institute for Physical Science and Technology, University of Maryland, College Park, MD 20742, USA
[3]Department of Physiology, University of Maryland School of Medicine, Baltimore, MD 21201, USA
[4]Department of Physics, University of Maryland, College Park, MD 20742, USA
*Corresponding author: SSMartin@som.umaryland.edu

Supplementary Video S1. **MCF10A and MDA-MB-231 cells exhibit distinct collective migration behavior.** MCF10A (left) and MDA-MB-231 (right) cell sheets migrating over 12 hours. Scale bars are 100 µm and clocks are shown as HH:MM.

Supplementary Video S2. Collective migration changes across a genetically defined cancer model system. From left to right: MCF10A, PTEN$^{-/-}$, KRas and KRas/PTEN$^{-/-}$ cell sheets migrating over 12 hours. Scale bars are 100 µm and clocks are shown as HH:MM.

Supplementary Dataset S1. Underlying Figure Data. Supplementary Data S1 contains tabular source data for all bar graphs, histograms, and curves in Fig. 1-3, Supplementary Fig. S2-S5, and Supplementary Fig S7 as well as the t-statistics in Fig. 4.

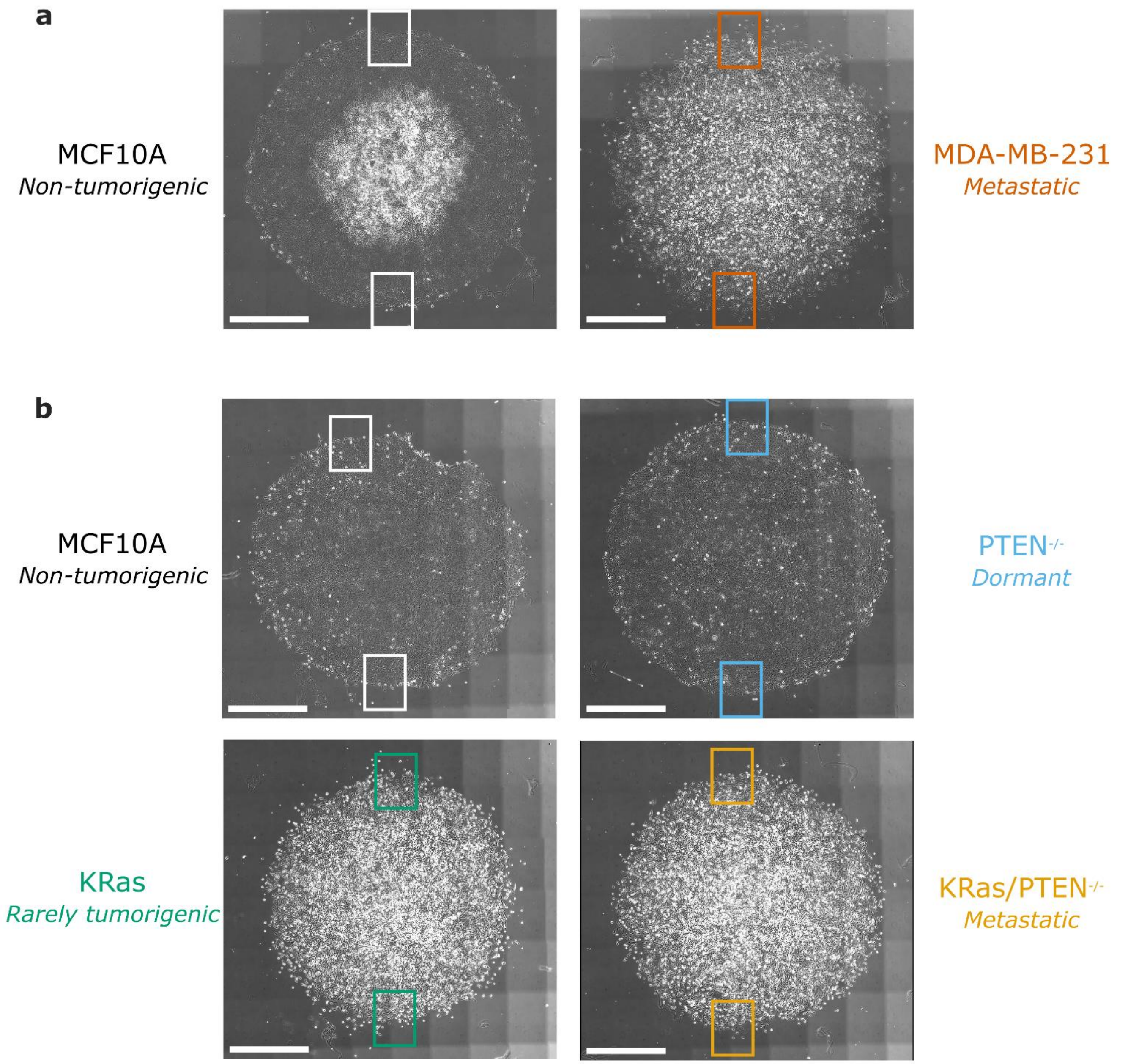

Supplementary Figure S1. Confluent, circular cell sheets are used for the collective migration assay. These tiled, phase contrast images show the entire circular cell sheet used for a collective migration assay. Time lapse images were taken of regions of interest (ROIs) at the top and bottom of each cell sheet for migration analysis. Approximate location of the ROIs for migration analysis are indicated by two rectangles on each image. Scale bars are 1 mm. **(a)** Images compare example MCF10A (left) and MDA-MB-231 (right) cell sheets. Regions at the bottom of these cell sheets were recorded over time to create **Supplementary Video S1**. **(b)** Images compare cell sheets from a genetically defined cancer model system. Regions at the bottom of these cell sheets were recorded over time to create **Supplementary Video S2**.

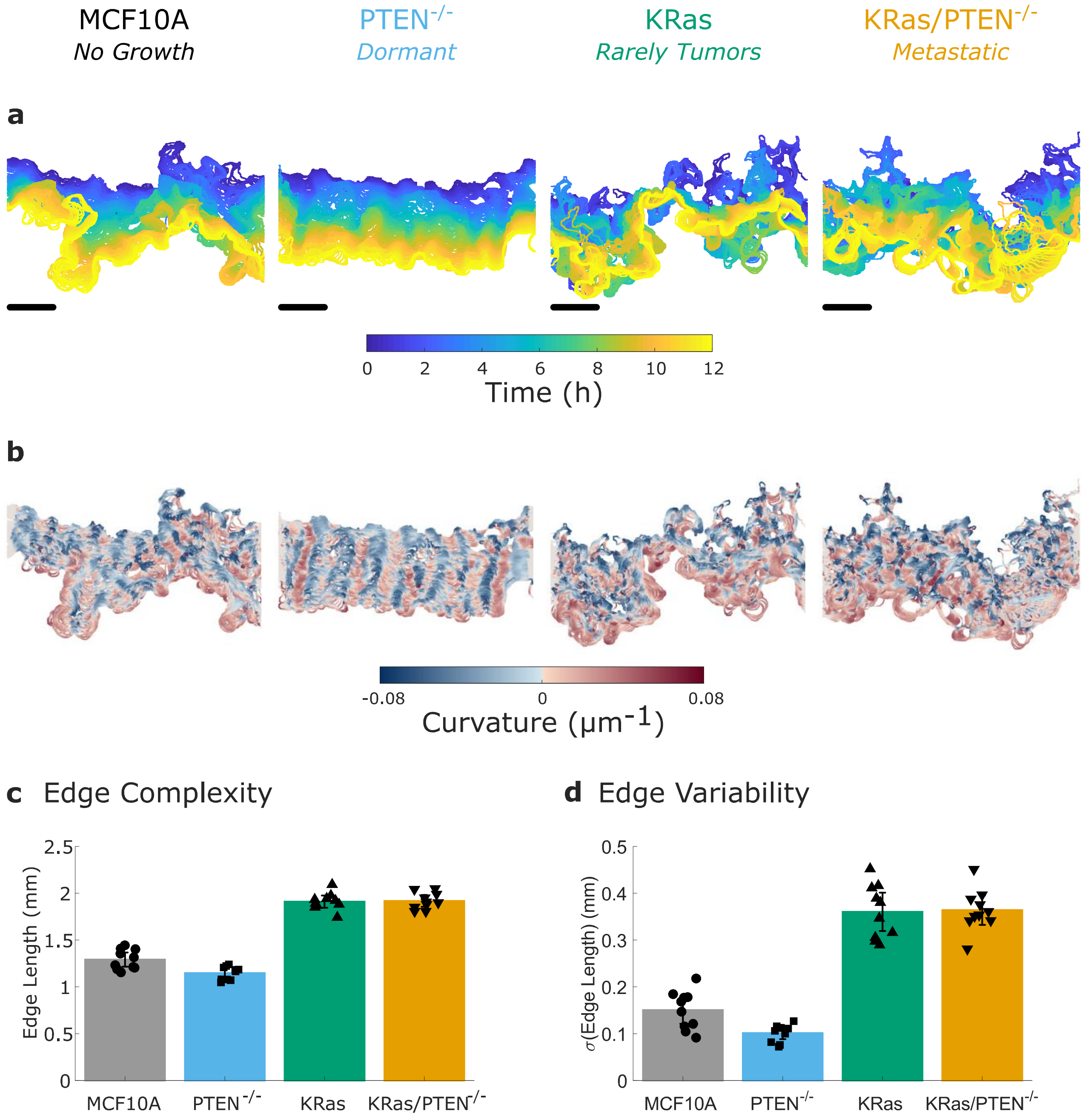

Supplementary Figure S2. PTEN$^{-/-}$ and KRas change edge dynamics. (a) The dynamics of the leading edge are shown by overlaid edges colored by time. **(b)** Coloring the leading edge by curvature illustrates the persistence or lack of persistence in local features of the edge shape. **(c)** Edge length is used to quantify the complexity of the leading edge. **(d)** The variability in edge length over time is used to quantify the dynamics of the leading edge. N = 10 independent experiments. Error bars indicate 95% confidence interval.

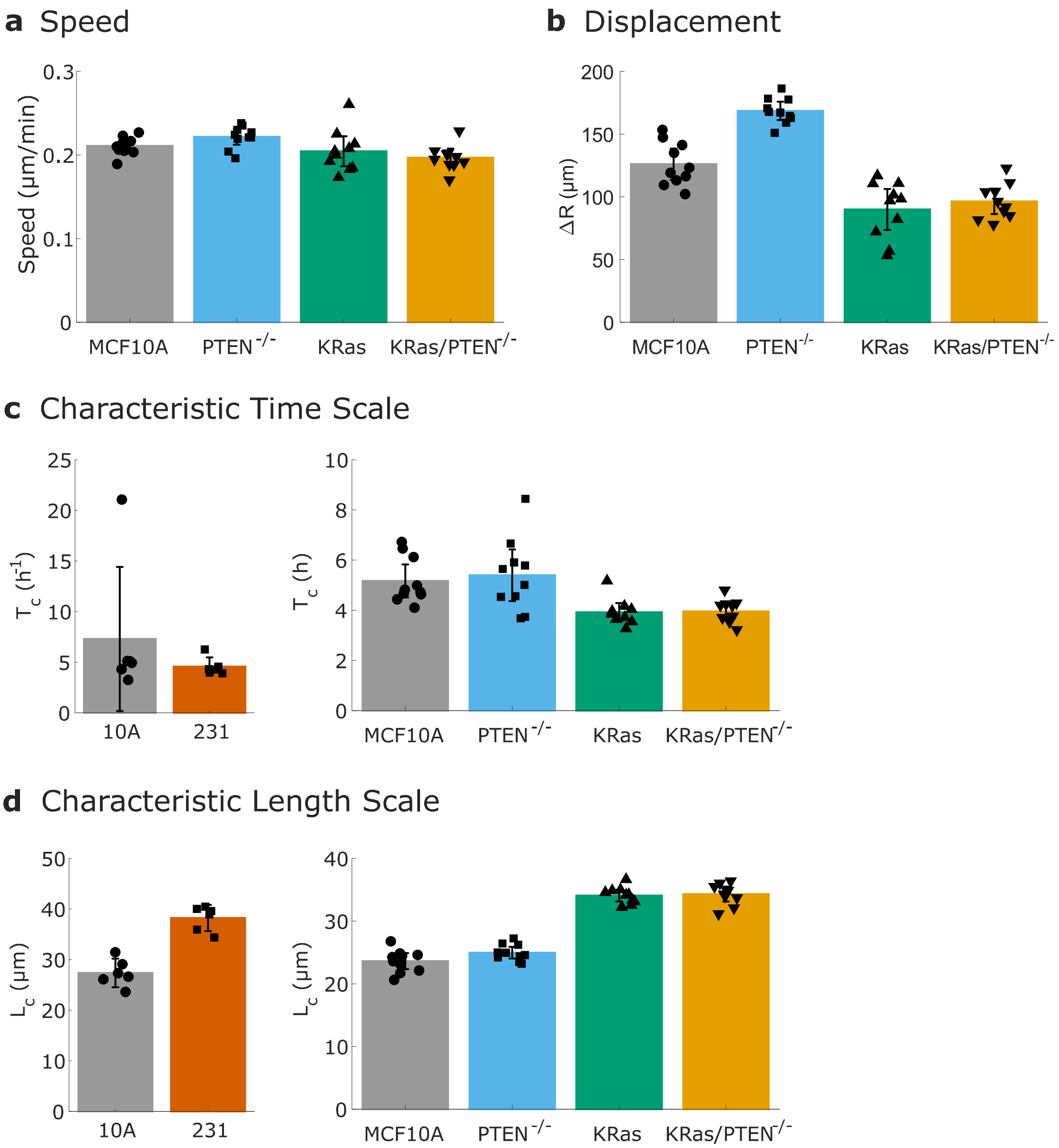

Supplementary Figure S3. Additional metrics enhance a multidimensional collective migration phenotype. **(a)** Mean speed of the PIV flow field. **(b)** Displacement of the leading edge. **(c)** Characteristic time scale of migration calculated using a coarse graining approach. **(d)** Characteristic length scale of migration calculated using a coarse graining approach. N = 10 (PTEN$^{-/-}$ and KRas) or N = 6 (231) independent experiments. Error bars indicate 95% confidence interval.

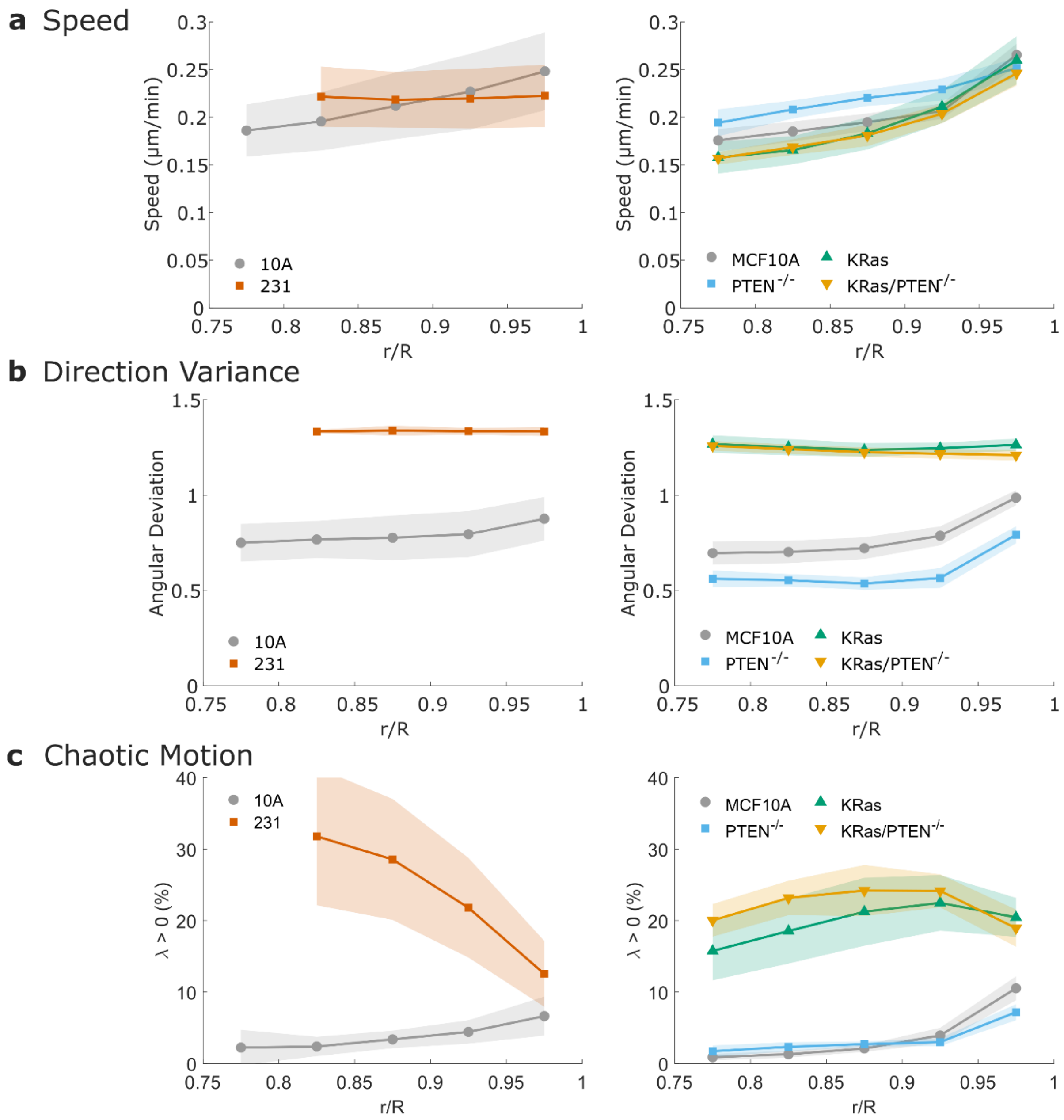

Supplementary Figure S4. Collective migration behavior varies as a function of distance from the leading edge. **(a)** Speed, **(b)** direction variance, and **(c)** chaotic motion are reported as a function of distance from the leading edge for MCF10A vs MDA-MB-231 (left) and a genetically defined cancer model system (right). In all figures, the location within the cell sheet, *r*, is normalized by the radius of the cell sheet, *R*. This results in an x-axis where *r/R* = 1 indicates the leading edge of the cell sheet, while 0 indicates the center of the cell sheet. Due to the ROIs used for time lapse imaging, metrics are not reported for *r/R* < 0.75. Data was binned using radial sections of size 0.05 *r/R*. Shaded regions indicate 95% confidence intervals.

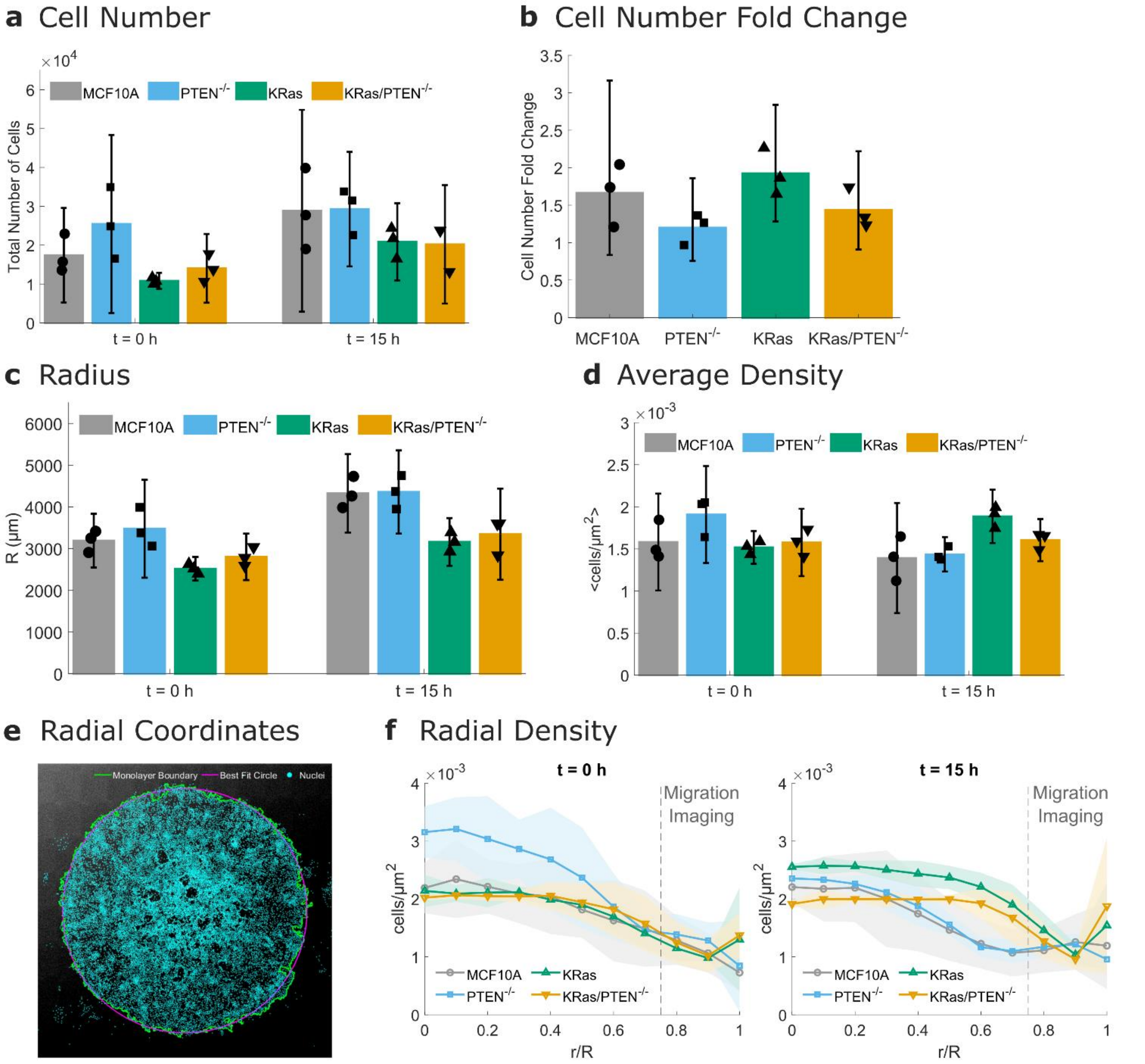

Supplementary Figure S5. Cell sheet density is similar across cell lines. Density measurements are based on counted nuclei (see **Supplementary Figure S6**). **(a)** Absolute cell numbers vary, but **(b)** the fold change in cell number over 15 h is similar. **(c)** Cell sheets show a trend towards larger radii in the MCF10A and $PTEN^{-/-}$ cell lines, which agrees with the displacement trends shown in the main text. **(d)** Average cell density is similar across cell lines. **(e)** Segmentation was used to create a best fit circle. **(f)** Cell density is measured as a function of *r/R*, shown at t = 0 h (left) and t = 15 h (right). Density is similar across cell lines, especially in the region used for collective migration imaging (*r/R* > 0.75). At t = 15 h, the MCF10A and $PTEN^{-/-}$ cell lines show lower densities further away from the edge, which is consistent with their collective migration behavior. Collective behavior leads to increased directional motion propagating away from the leading edge, and thus more *r/R* regions contribute to migration. These regions spread out and have decreased density. N = 3 independent experiments. Error bars or shaded regions indicate 95% confidence intervals.

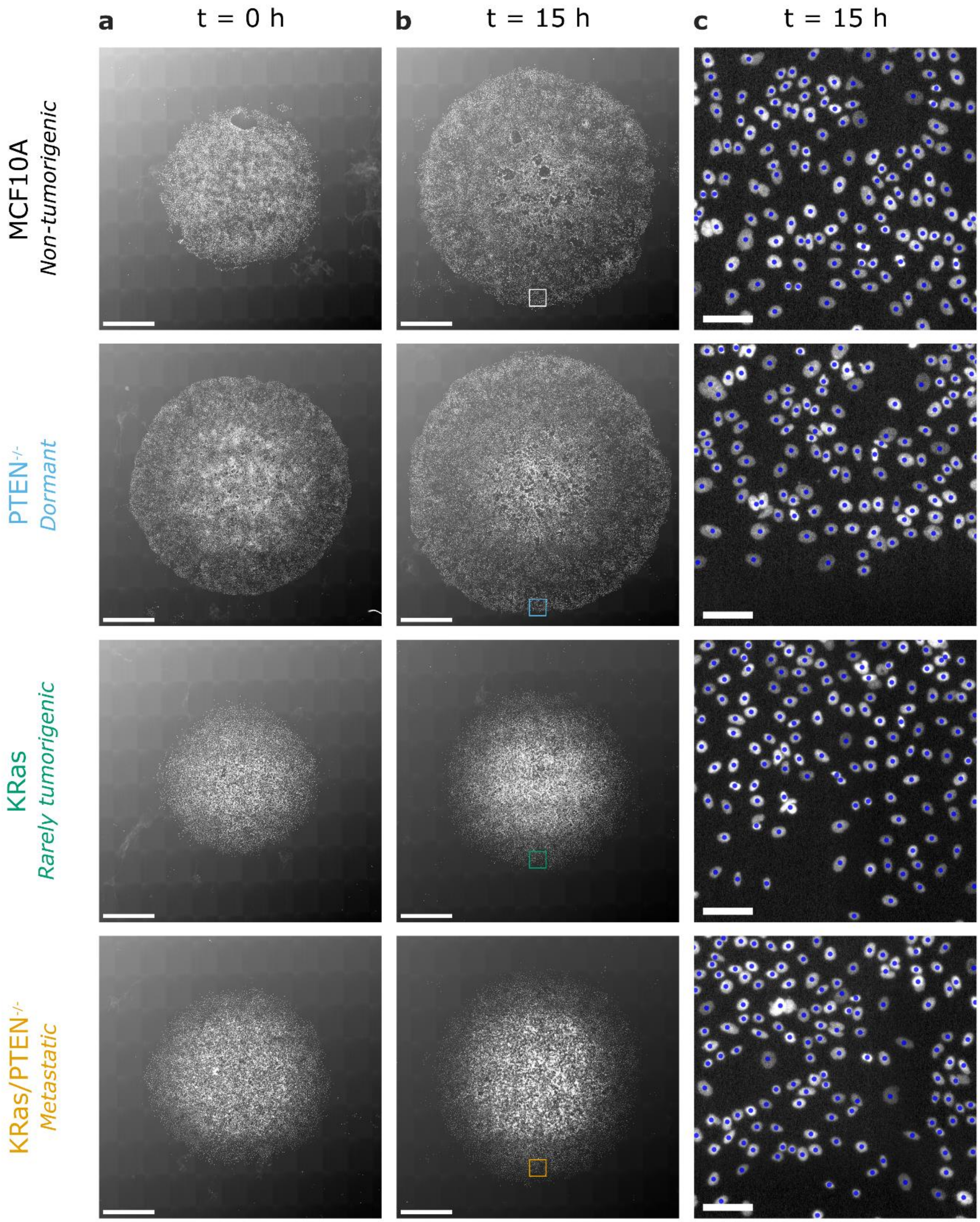

Supplementary Figure S6. DAPI staining was used to measure cell density. Duplicate sets of cell sheets were fixed at time points corresponding to the start of migration and end of migration. Cell sheets were stained with DAPI and individual nuclei were counted using a peak finding algorithm (see **Methods**). DAPI staining of tiled monolayers is shown at **(a)** t = 0 h and **(b)** t = 15 h. Scale bars in **(a,b)** are 1 mm. **(c)** ROIs at the edge of the cell sheet show blue dots on each counted nucleus. Scale bars in **(c)** are 50 µm. Representative images from one experiment; N = 3 independent experiments were conducted.

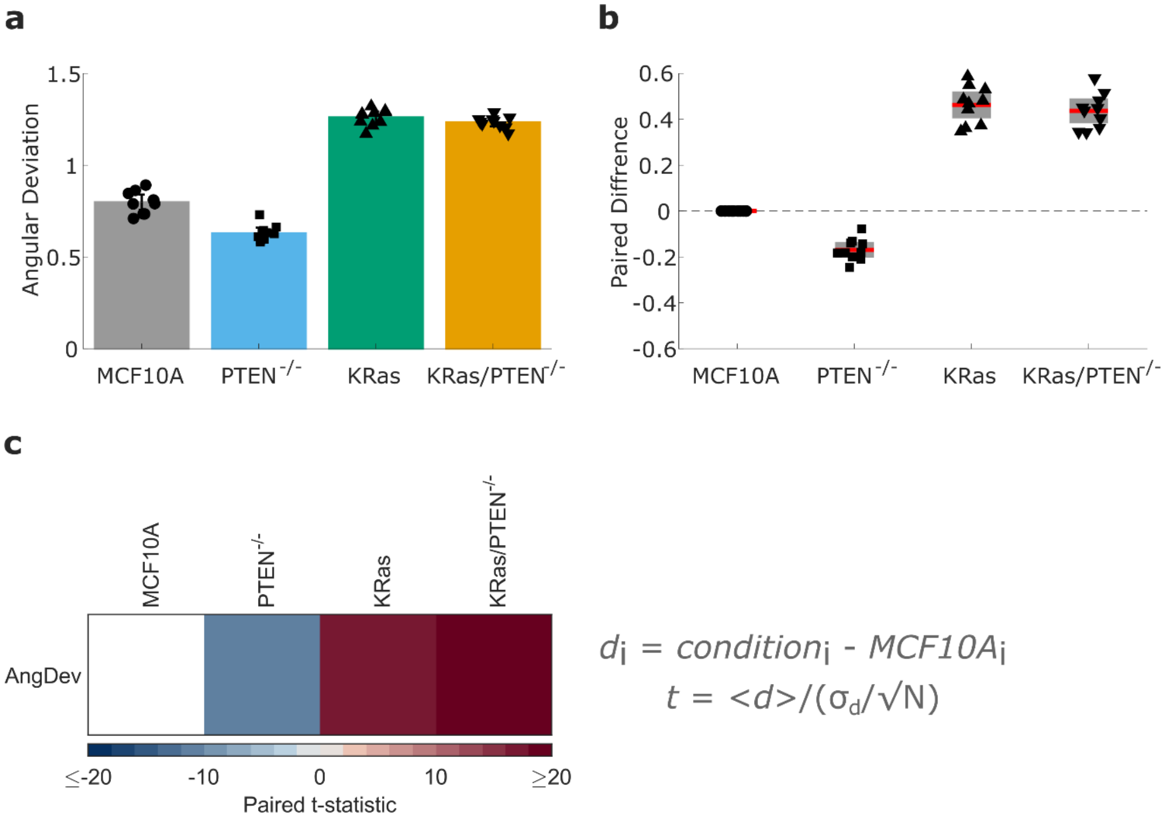

Supplementary Figure S7. Paired statistics were used for clustering analysis. **(a)** Variability in velocity direction quantified by angular deviation (as shown in **Fig. 3C**). **(b)** Independent experiments were conducted using paired migration assays, allowing for the calculation of the paired difference for each cell line compared to the same-experiment MCF10A control. **(c)** These paired differences can be used to calculate to a paired t-statistic. The paired t-statistic is calculated as the mean of the differences (<*d*>) divided by the standard error of the differences (standard deviation of the differences, σ_d, divided by the square root of the number of independent experiments, *N*). This allows for the comparison of the strength of changes across metrics which may be measured in different units and on different scales. N = 10 independent experiments. Error bars indicate 95% confidence interval.
